# Methods of Measuring Mitochondrial Potassium Channels: A Critical Assessment

**DOI:** 10.3390/ijms23031210

**Published:** 2022-01-21

**Authors:** Agnieszka Walewska, Milena Krajewska, Aleksandra Stefanowska, Aleksandra Buta, Renata Bilewicz, Paweł Krysiński, Piotr Bednarczyk, Piotr Koprowski, Adam Szewczyk

**Affiliations:** 1 Laboratory of Intracellular Ion Channels, Nencki Institute of Experimental Biology, Polish Academy of Sciences, 02-093 Warsaw, Poland; a.walewska@nencki.edu.pl (A.W.); m.krajewska@nencki.edu.pl (M.K.); aa.stefanowska@gmail.com (A.S.); abuta@chem.uw.edu.pl (A.B.); 2 Faculty of Chemistry, University of Warsaw, 02-093 Warsaw, Poland; bilewicz@chem.uw.edu.pl (R.B.); pakrys@chem.uw.edu.pl (P.K.); 3 Department of Physics and Biophysics, Institute of Biology, Warsaw University of Life Sciences-SGGW, 02-776 Warsaw, Poland; piotr_bednarczyk@sggw.edu.pl

**Keywords:** mitochondria, potassium channels, patch-clamp, planar lipid bilayer, solid supported membranes, cubic phases

## Abstract

In this paper, the techniques used to study the function of mitochondrial potassium channels are critically reviewed. The majority of these techniques have been known for many years as a result of research on plasma membrane ion channels. Hence, in this review, we focus on the critical evaluation of techniques used in the studies of mitochondrial potassium channels, describing their advantages and limitations. Functional analysis of mitochondrial potassium channels in comparison to that of plasmalemmal channels presents additional experimental challenges. The reliability of functional studies of mitochondrial potassium channels is often affected by the need to isolate mitochondria and by functional properties of mitochondria such as respiration, metabolic activity, swelling capacity, or high electrical potential. Three types of techniques are critically evaluated: electrophysiological techniques, potassium flux measurements, and biochemical techniques related to potassium flux measurements. Finally, new possible approaches to the study of the function of mitochondrial potassium channels are presented. We hope that this review will assist researchers in selecting reliable methods for studying, e.g., the effects of drugs on mitochondrial potassium channel function. Additionally, this review should aid in the critical evaluation of the results reported in various articles on mitochondrial potassium channels.

## 1. Introduction

Ion channels are present in all intracellular membranes [1,2,3]. When compared to plasma membrane channels, functional studies of these channels present new experimental challenges and difficulties. First, purification of intracellular membranes is often necessary before functional studies. Membrane purification is accomplished by well-known techniques, but the high purity of the final preparations poses a considerable challenge. This is especially true when the final functional assay is based on single-channel activity measurements. The limited purity of the final membrane preparations is due to not only the technical limitations of the applied procedure but also in vivo cross-interactions of various intracellular membranes [4]. The continuity of the endoplasmic reticulum with the nuclear membrane is a clear example of such a problem. Second, the unique microenvironment (both functionally and structurally) of intracellular compartments (including mitochondria) affects in vitro studies of various ion channels [5,6,7]. These issues will be discussed in greater detail later in this paper.

All of the pitfalls discussed above are observed in functional studies of mitochondrial potassium channels present in inner mitochondrial membranes. Moreover, the density of mitochondrial potassium channels is very low, indicating that they are low-copy-number proteins [8,9]. The similarity of the mitochondrial channels to those in the plasma membrane aids in the selection of specific drugs affecting channel activity [10,11,12]. However, at the same time, researchers must precisely design experiments to ensure that mitochondrial potassium channels, rather than the plasma membrane channels, are studied.

**Figure 1:**
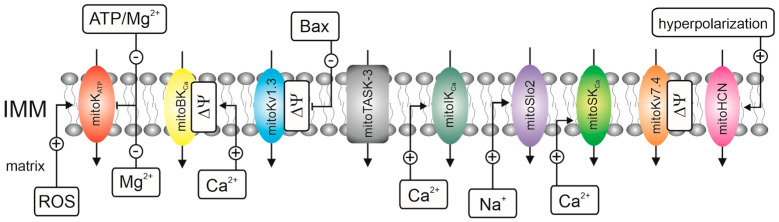
Potassium channels present in the inner mitochondrial membrane with their endogenous modulators. From the left: ATP-regulated (mitoK_ATP_) channel, large-conductance Ca^2+^-regulated (mitoBK_Ca_) channel, voltage-gated (mitoKv1.3) channel, two-pore domain (mitoTASK-3) channel, intermediate-conductance Ca^2+^-regulated (mitoIK_Ca_) channel, large-conductance Na-regulated (mitoSLO2) channel, small-conductance Ca^2+^-regulated (mitoSK_Ca_) channel, voltage-gated (mitoKv7.4) channel, and hyperpolarization-activated and cyclic nucleotide-gated (mitoHCN) potassium channel.

Mitochondria are essential for cellular metabolism and energy production [13]. ATP synthesis is driven by proton motive force across the inner membrane potential playing a major role. The high membrane potential of the inner mitochondrial membrane is the main parameter of efficient ATP synthesis, as described by Peter Mitchell. This property indicates that the inner membrane is an insulator or has low permeability to charged substances. A plethora of inner membrane transporting proteins has been described in the inner mitochondrial membrane [14]. These include anion- and cation-selective ion channels as well as transport proteins that affect membrane potential, such as ATP/ADP translocase [15,16]. The following potassium channels were identified in the inner mitochondrial membrane: mitochondrial ATP-sensitive potassium (mitoK_ATP_) channels [17,18,19,20], mitochondrial large-conductance calcium-activated potassium (mitoBK_Ca_) channels [21,22,23], mitochondrial intermediate-conductance calcium-activated potassium (mitoIK_Ca_) channels [24,25], mitochondrial small-conductance calcium-activated potassium (mitoSK_Ca_) channels [26,27], mitochondrial sodium-activated potassium (mitoSLO2) channels [28], mitochondrial voltage-regulated potassium (mitoKv) channels [29,30], and mitochondrial double-pore potassium (mitoTASK) channels [31,32] (Figure 1). The physiological significance of mitochondrial potassium channels in cell proliferation and death signaling is well documented [5,33,34,35].

Based on biophysical and pharmacological properties, one could claim the similarity of the plasma membrane to mitochondrial potassium channels. In recent years, the molecular identity of mitochondrial potassium channels has been partially established [24,25]. However, some issues have not been clarified and must be studied, including the cytoprotection mechanism induced by mitochondrial potassium channel activation, specific isoforms of mitoBK_Ca_ channels in various tissues, the identification of mitochondrial auxiliary potassium channel subunits, and the final molecular identification of mitoK_ATP_ channels [36,37].

The environment in which mitochondrial potassium channels operate differs from that of the plasma membrane. This unique environment concerns both membrane lipids and protein vicinity. It directly affects the basic functions of mitochondrial potassium channels. Additionally, the active role of mitochondria as the site of reactive oxygen species (ROS) synthesis differs considerably from that of the plasma membrane. In addition, the protein neighborhood in mitochondria serves as a specific location for mitochondrial potassium channels. For example, it was shown that mitoBK_Ca_ channels have structural–functional interactions with respiratory chain proteins [38].

Potassium channels in the inner mitochondrial membrane are modulated by inhibitors (blockers) and activators (potassium channel openers) that have been previously described for potassium channels in the plasma membrane. Unfortunately, the majority of mitochondrial potassium channel modulators exhibit a wide range of off-target effects. These include uncoupling properties, respiratory chain inhibition, and effects on cellular calcium homeostasis. Therefore, the correct application of potassium channel inhibitors or activators is crucial in functional studies of intact cells. This important problem has been previously reviewed in detail [12,39,40,41].

In this paper, we present a critical review of methods used to study the functions of mitochondrial potassium channels. These methods can be classified into three groups:

- Electrophysiological techniques, i.e., the planar lipid bilayer (PLB) technique and patch-clamp technique;
- Flux K^+^ measurements that use fluorescent probes, i.e., small molecule probes, genetically encoded probes, and thallium (Tl^+^)-sensitive indicators. We omit flux measurements that use rubidium cations as a tracer for K^+^. The use of radioactive 86Rb^+^ and 87Rb^+^ to study mitochondrial potassium channels is uncommon [42] despite the widespread use of 86Rb^+^ to study plasma membrane and intracellular potassium channels [43];
- Biochemical techniques, i.e., the use of unique mitochondrial properties such as swelling, respiration, and high membrane potential to track potassium fluxes via inner membrane channel proteins.

A synopsis of the techniques described in this review can be found in Table 1. The contribution of these methods for advancing our knowledge on mitochondrial K^+^ channels is quite complex. Electrophysiological techniques such as patch-clamp or PLB allowed characterizing single-channel activities of mitochondrial potassium channels. This helped not only to determine biophysical properties of the channels such as conductance or selectivity but was crucial for describing mitochondrial potassium channel pharmacology. Potassium cation flux measurements with the use of fluorescent probes helped to characterize K^+^ traffic in intact cells. Finally, biochemical techniques contributed to our understanding of the functional role of mitochondrial potassium channels.

At the end of this paper, we will provide some basic information on the potential use of other techniques, such as solid supported membranes or the application of lipid liquid crystalline cubic phases, to study the function of mitochondrial potassium channels.

## 2. Reconstitution of Mitochondrial K^+^ Channels into Planar Lipid Bilayers

**Figure 2:**
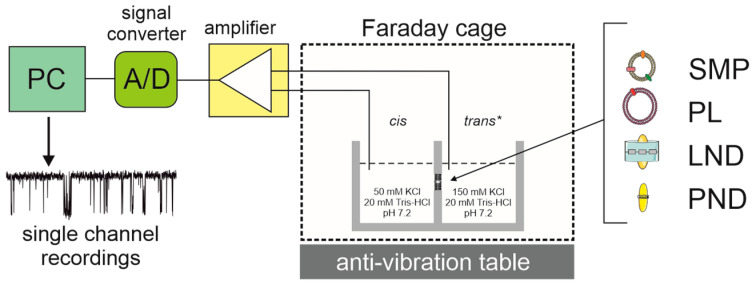
Planar lipid bilayers (PLBs) were used to record mitochondrial K^+^ channel activity. The main PLB equipment includes a personal computer (PC) with software, a signal converter (A/D), an amplifier, a Faraday cage, an antivibration table, and a holder with separate cis and trans compartments (*—grounded site). Alternative systems to incorporate channels to PLB include: SMP—submitochondrial particles, PL—proteoliposomes, LND—lipid nanodiscs (MSP1- and SMA-based nanodiscs), and PND—peptidiscs (peptide-based nanodiscs).

The planar lipid bilayer (PLB) technique, also known as the black lipid membrane (BLM) technique, was first described by Muller in 1962 [78]. This well-established method has been extensively used in recent decades to study a plethora of ion channels, including those found in intracellular membranes [44,45]. This is due to the inherent characteristics of the PLB technique. First, an artificial lipid membrane is formed across a small diameter (150 to 250 µm) cavity in a sheet of Teflon, Delrin, or polystyrene. Next, a suspension of channel-containing membrane vesicles is added and, after the ion channel is spontaneously incorporated into the lipid bilayer, the ion flow is measured as the change in current over time. The PLB technique has been used to identify and characterize mitochondrial channels from various tissues and organisms [39] (Figure 2). In this case, mitochondria are isolated, and submitochondrial particles (SMPs) are prepared by exposing the mitochondria to ultrasound or osmotic shock. In the next step, SMPs are fused to artificial membranes. The activity of the mitochondrial ATP-sensitive potassium (mitoK_ATP_) channel from cardiac mitochondria has been intensively studied [46,47,48,49].

The ability to record channel activity on a single-molecule level under fully controlled conditions distinguishes the PLB technique from the patch-clamp technique. It should be noted that the lipid environment in PLB is artificial and different lipid environments can impact channel gating behavior. Usually, in PLB experiments lipid bilayers are formed with soybean asolectin due to its low price, ease in forming bilayers, and complex lipid composition [20,36]. Soybean asolectin contains phosphatidylcholine (PC), phosphatidylethanolamine (PE), and phosphatidylinositol (PI), each accounting for ca. 25% of total lipids, along with minor amounts of other phospholipids, which is significantly different from the lipid content of IMM. PC and PE are still the most abundant phospholipids in the IMM, comprising up to 75% of total lipids. However, the third most abundant and the most unique lipid for IMM is cardiolipin (CL) which makes up about 15–20% of the total phospholipids. Minor lipids are phosphatidylserine (PS), 3%, and PI, 5% [79]. The PLB technique is particularly useful for investigating the role of lipid constituents in channel gating, and in the case of mitochondrial channels it would be favorable to mimic the specific lipid environment of the IMM. However, the impact of the specific lipid environment of the IMM on potassium channels has not been studied to date. The PLB technique also provides easy access to both sides of the channel after incorporation, which allows for investigating the topology of ion channel–drug binding. Due to these qualities of the PLB technique, properties of the cardiac mitoK_ATP_ channel, such as the ATP, pH, or Mg^2+^ regulation, have been reported [46,47]. One serious drawback associated with the identification of mitochondrial channels by PLB measurements is the purity of the mitochondrial fraction. Because PLB recordings have a single-molecule resolution, even minor contamination could lead to false conclusions. Similarly, the use of SMPs does not allow for molecular identification of channel proteins based on electrophysiological activity due to their multiprotein composition. However, advances in protein purification, including the introduction of new detergents, aid in identifying the molecules responsible for well-known mitochondrial channel activities. In this way, ATP synthase was purified to near homogeneity, and it was shown that ATP synthase dimers, but not monomers, are responsible for the so-called “megachannel” activity [80]; using a similar technique, however, it was shown that monomeric ATP synthase also formed the same “megachannel” activity [81]. As an alternative, complementary, e.g., biochemical, methods could be used to identify molecular candidates for mitochondrial channels, and the electric activity of recombinant purified proteins could then be tested in PLBs. The CCDC51 protein was proposed based on its mitochondrial localization and topology as a pore constituent of the mitoK_ATP_ channel and renamed MITOK. Recombinant MITOK-6xHIS protein was expressed either in *Escherichia coli* or by coupled in vitro transcription/translation, purified, and reconstituted in liposomes, and its electric activity was detected and characterized in PLBs [36].

In recent years, nanodisc technology has revolutionized membrane protein science [82]. All nanodiscs are soluble fragments of lipid bilayers (lipid nanodiscs (LNDs)), but the way these membrane fragments are stabilized may differ. Thus, there are two main types of nanodiscs: those made of belt proteins, such as membrane scaffold protein (MSP) derivatives [83], and those made of amphipathic copolymers, such as styrene–maleic acid (SMA) [84]. In addition to lipid nanodiscs, which contain membrane fragments, membrane proteins could be stabilized in solution by amphipathic peptides, which form so-called peptidiscs (peptide nanodiscs (PNDs)) without the involvement of detergents and lipids [85,86]. However, to date, PNDs have not been used to deliver channels into PLBs.

To incorporate membrane proteins into MSP nanodiscs, proteins from native membranes must be solubilized and reconstituted into self-assembling MSP nanodiscs [87]. However, membrane proteins can also be directly incorporated into MSP nanodiscs during translation. For instance, viral ion channels have been incorporated into MSP nanodiscs during coupled in vitro transcription/translation reactions and then efficiently transferred into a PLB, allowing recording of ion channel activity [88]. This approach allows for the study of proteins while also providing a fast, reliable, and contamination-free method for the functional analysis of ion channels, eliminating the need to obtain material from living cells.

Amphipathic copolymers such as SMA exhibit detergent-like properties and can form nanodiscs via direct membrane solubilization without the use of detergents. Therefore, SMA nanodiscs are often referred to as “native nanodiscs” [89]. SMA was used to solubilize yeast mitochondrial respiratory complex IV, showing that large membrane protein complexes could be stabilized within these particles [90]. Moreover, artificial mitochondrial membranes could be formed within SMA nanodiscs [91]. Despite the potential benefits of nanodiscs, only one potential mitochondrial channel isoform, ROMK2, expressed in *Escherichia coli* cells, has been investigated with this technology [50]. In this study, channel activity was observed more often when the ROMK2 channel was reconstituted in nanodiscs than when it was reconstituted into proteoliposomes before adding to PLBs, suggesting an advantage of nanodisc technology.

In conclusion, the well-established PLB technique currently provides a fully controlled environment (both in terms of protein and lipid composition) for the reconstitution of multi-subunit mitochondrial potassium channels, indicating further use of this technique in the future.

## 3. Patch-Clamp of the Inner Mitochondrial Membrane

The patch-clamp technique was developed by Erwin Neher and Bert Sakmann more than 40 years ago [92,93] and is currently one of the most important electrophysiological methods for recording the activity of ion channels in their native environment. The patch-clamp technique has been modified to study ionic currents in tissue sections [94]; organelles such as lysosomes [95,96], mitochondria [38,51,97], and nuclei [98,99]; and bacteria [100,101].

**Figure 3:**
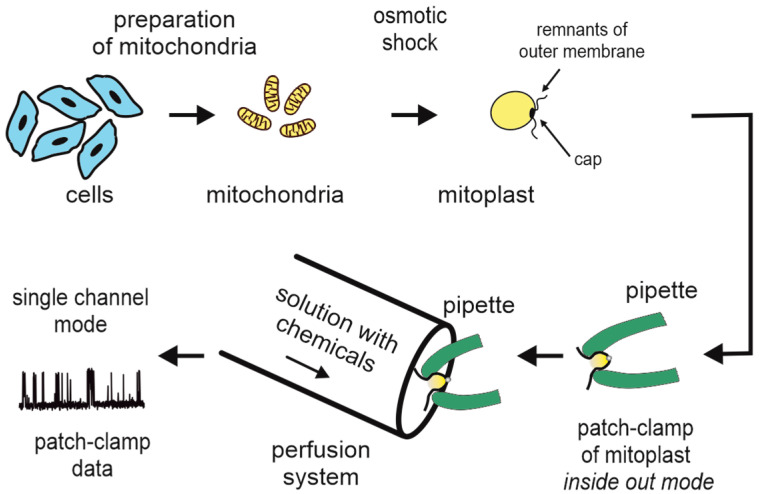
Patch-clamp technique in mitochondrial K^+^ channel studies. Schematic representation of the preparation of mitochondria and mitoplasts and the patch-clamp experiment in inside-out mode with a perfusion system.

One specific application of the patch-clamp technique is the recording of the electric activity of ion channels and transporters residing in the inner mitochondrial membrane, which was pioneered by Sorgato et al. [97] and quickly followed by Petronilli [51]. With the patch-clamp technique, mitochondrial potassium channels were discovered in human bronchial epithelial cells [52], skin fibroblasts [53], cardiomyocytes [54], endothelial cells [23], keratinocytes [55], astrocytomas [38], and lymphocytes [29]. Thanks to this technique, many different types of channels have been discovered in the inner mitochondrial membrane, including potassium channels inhibited by ATP (mitoK_ATP_) [56,57], intermediate-conductance calcium-regulated potassium channels (mitoIK_Ca_) [24], large-conductance calcium-activated potassium channels (mitoBK_Ca_) [21], and mitochondrial megachannels (also known as permeability transition pores) (mPTP) [102] (Figure 3). The most important advantage of the patch-clamp technique is the ability to record the activity of ion channels in their native environment, namely, in the inner mitochondrial membrane. In addition, this technique enabled researchers to investigate the regulation of mitochondrial ion channel activity by various substances (natural origin as well as synthetic modulators of channel activity). New regulatory mechanisms of the activity of mitochondrial ion channels have been discovered. For instance, it has been shown that mitoBK_Ca_ channels are regulated by membrane stretch [103], gasotransmitters [34], or the natural flavonoids naringenin [104] and quercetin [105]. Due to the above-mentioned advantages and relatively high frequency of recording channel activity, patch-clamp of the IMM appears to be a convenient technique for studying mitochondrial ion channels and is recognized as the gold standard. Therefore, the potential drawbacks of this technique determine the quality of the data and the advancements of the research field.

A patch-clamp technique variant that uses a concentric electrode arrangement in which an external pipette protects an inner patch pipette during plasma membrane penetration, after which a seal can be formed on an internal organelle membrane, e.g., a mitochondrial membrane, has previously been described [106]. However, this technique is complicated [107]. Therefore, to study the activity of mitochondrial channels by patch-clamp, mitochondria must be isolated. In general, freshly isolated mitochondria are used; therefore, the time spent on their isolation is kept to a minimum, and simple differential centrifugation is used to obtain the mitochondria-enriched fraction [108]. However, in some studies, an additional Percoll gradient purification step was added to increase mitochondrial purity [28]. Mitochondria are surrounded by two membranes: a smooth outer membrane (OMM) and a folded inner mitochondrial membrane (IMM). To record channel activity from the IMM, the OMM is disrupted using hypoosmotic shock to form mitoplasts. Mitoplasts are recognized as transparent circular objects with characteristic dark caps formed by OMM remnants that are visible under a phase-contrast microscope. The rest of the method is straightforward. After capturing a mitoplast with a recording pipette under negative pressure (suction) and gigaohm seal formation, a membrane patch either forms spontaneously at the tip of the pipette or is excised by tapping the pipette holder. Thus, all that is left to do is record the channel activity. However, when patching mitoplasts, the first source of uncertainty is the purity of the isolated mitochondria and the choice of vesicles identified as mitoplasts among other cellular debris. Despite its characteristic shape and cap, distinguishing a mitoplast from other objects may be challenging for inexperienced researchers. Mitochondria containing DNA (mtDNA) allowed for the development of a single-mitoplast PCR assay [74], which can confirm that the selected object is a mitoplast. In this assay, a single intact mitoplast was aspirated by a glass pipette with a tip large enough to accommodate the mitoplast and was then transferred into a PCR tube. To detect mtDNA, PCR with primers that can specifically amplify a fragment of mtDNA was used. The separation and visualization of the PCR product by standard agarose gel electrophoresis proves that the acquired vesicle was a mitoplast. In this way, the correct objects can be selected for the patch-clamp technique, despite the presence of cellular debris that remains after mitochondrial isolation. In a more direct approach, mitochondria can be labeled with a fluorescent protein (e.g., YFP) that is specifically targeted to mitochondria [109] or loaded with a mitochondria-specific fluorescent dye (e.g., MitoTracker) that is trapped inside mitochondria during purification and swelling [77]. Then, fluorescent mitoplasts can be selected under a fluorescence microscope during the patch-clamp experiment. This direct method appears to be the most advantageous for identifying the correct object—the mitoplast.

However, even these methods cannot guarantee that the activity of channels from the IMM is recorded. It is assumed that the unique mitoplast morphology is due to the expansion of the folded IMM, the rupture of the OMM, and the disintegration of the remaining intracellular organelles. Interestingly, mitochondria have a molecular relationship with the endoplasmic reticulum (ER), which results in the presence of mitochondria-associated membranes (MAMs). It has been shown that in cells subjected to hypotonic shocks, all organelles were fragmented and swollen into vesicles [110]. However, at the same time, interorganellar contacts were preserved, allowing vesicle pairs of swollen mitochondria and MAMs to be observed. It is therefore plausible that MAMs rather than IMMs could be patched. For instance, based on subcellular fractionation, ryanodine receptors (RyRs) were assumed to be present in IMMs, and their supposed mitochondrial localization was supported by confocal microscopy [111]. Mitochondrial localization of RyRs was further favored by patch-clamp recordings on mitoplasts [112]. In these recordings, only 1.1% of patches had RyR channel activity. However, Noterman et al. [113], based on an improved mitochondrial fractionation protocol, recently ruled out the presence of RyRs in mitochondria. It can therefore be speculated that the small number of RyR channels active in membrane patches derived from objects identified as mitoplasts were, in fact, present not in IMMs but in MAMs.

What are some possible solutions to such ambiguities? First, patch-clamp experiments could be carried out not only in the excised-patch configuration but also in the whole-mitoplast configuration [109,114]. In this way, all the activities present in the vesicle are recorded, and single channels that “get in the way” and are interpreted as mitochondrial are not counted. Second, in the excised-patch configuration, characteristic channel activity found only in mitochondria can be used as an internal standard. This type of activity, i.e., mitochondrial megachannel, was identified in early patch-clamp studies on mitoplasts [102]. The pharmacology of the mitochondrial megachannels is well known [115,116], and current research suggests that ATP synthase dimers or monomers might be the main molecular component [82,117]. Because megachannels can be activated or inhibited by several compounds [118] and detected in the majority of IMM patches [119], their activity can be correlated with the activity of a channel under investigation. To our best knowledge, only Szabo et al. [29] used megachannel activity induced by the addition of 0.1 mM Ca^2+^ as a marker of IMM for establishing mitochondrial localization of the Kv1.3 channels.

The next disadvantage of patch-clamp recordings, whether in the excised-patch or the whole-mitoplast configuration, is that the inner mitochondrial membrane is disrupted during patch formation, resulting in the loss of soluble mitochondrial matrix components. To mitigate this phenomenon, one can use a perforated-patch configuration [120]. However, no studies on mitoplasts in this configuration have been carried out.

Despite the problems mentioned above, patch-clamp of the inner mitochondrial membrane is a unique approach for studying ion channel activity that has significantly contributed to the advancement of the field of mitochondrial potassium channels.

## 4. K^+^-Specific Fluorescent Indicators in Mitochondrial Potassium Channel Studies

Reconstitution of mitochondrial potassium channels into PLBs and mitoplast patch-clamp experiments both require cell disruption and mitochondria purification. This is followed by the preparation of mitoplasts (in case of patch-clamp) or submitochondrial particles (in case of channel reconstitution into planar lipid bilayers). All these procedures introduce the methodological difficulties mentioned in previous sections. Hence, techniques for measuring mitochondrial potassium ion (and other alkali metal ion) trafficking have been intensively investigated for many years [58,59] with help of fluorescent indicators. Unfortunately, most of these fluorescent indicators suffer from limited specificity for K^+^, low dynamic range, difficulty of loading into cells, no selective targeting to mitochondria, and toxicity.

The optimal mitochondrial K^+^ sensor should have a dynamic range compatible with the intracellular K^+^ concentrations, i.e., 50–150 mM in different cell compartments. Additionally, it should be insensitive to pH and millimolar Na^+^ concentrations. Finally, it should be located in the mitochondrial matrix. This final property can be obtained relatively easily by attaching a lipophilic triphenylphosphonium cation (TPP^+^) to the sensor. Because of the negatively charged mitochondrial interior, sensors with TPP^+^ moiety will efficiently accumulate in the mitochondrial matrix.

Several small-molecule fluorescent K^+^ sensors have been developed to image K^+^ fluctuations using fluorescence microscopy [110]. The most well known K^+^ sensors for biological studies are based on crown ethers and include potassium-binding benzofuran isophthalate (PBFI) and its cell-permeable form bis(acetyloxymethyl) esterified PBFI (PBFI-AM), as well as Asante Potassium Green-1 (APG-1) [60,61]. Unfortunately, these probes suffer from poor selectivity for K^+^ over Na^+^ and difficult quantification of absolute [K^+^] and require excitation with phototoxic UV-light. Hence, PBFI was used to study the function of mitochondrial ATP-regulated potassium (mitoK_ATP_) channels in well-defined conditions following liposome reconstitution [62]. The same probe was used to study mitoBK_Ca_ activity in isolated cardiac mitochondria [63]. Interestingly, intracellular K^+^ fluxes were also measured in cultured hippocampal neurons by using PBFI to better understand the role of K^+^ in neuronal death under ischemic conditions [121].

A fluorescent K^+^ sensor targeted to mitochondria was produced by attaching TPP^+^ to triazacryptand-modified 3-styrylated BODIPY and designated KS6 [122]. It was found to be insensitive to pHs in the range of 5.4 to 9.0 as well as other metal cations. Localization of this probe was observed in the mitochondria of HeLa and U87MG cells [122]. A new mitochondrial-targeted K^+^ probe based on crown ethers, known as NK1, was recently synthesized [123]. NK1 has a good response to K^+^ but a poor response to pH changes and other metal cations and was used to measure K^+^ fluxes in the mitochondria of living cells. However, the fluxes measured by KS6 and NK1 were induced by ionophores (nigericin or ionomycin). Hence, both probes must be tested under more physiological conditions. Recently, the first near-infrared (NIR) fluorescent mitochondrial-targeted K^+^ probe was developed [124]. This probe contained a rhodamine analog as the fluorophore and triazacryptand as the K^+^-binding moiety. The efflux of K^+^ from mitochondria during apoptosis was reported by using this probe, taking advantage of the unique properties of the probe: a large Stokes shift (120 nm) and a long emission peak wavelength at 720 nm [124]. It should be noted that K^+^ flux may affect mitochondrial membrane potential and, as a result, the accumulation of the TPP^+^-conjugated probes. Therefore, carefully designed experimental schemes should be employed to avoid potential errors.

The use of thallium cations as K^+^ surrogates with Tl^+^-sensitive fluorescent indicators appears to be especially useful in functional studies of potassium channels. Because the ionic radii of Tl^+^ (0.154 nm) and K^+^ (0.144 nm) are similar, Tl^+^ is conducted by K^+^ channels [125]. The cells are loaded with nonfluorescent, thallium-specific dye while thallium ions flow through open potassium channels. Thallium cations bind the dye, generating a fluorescent signal proportional to channel activity. Benzothiazole coumarin acetoxymethyl ester (BTC-AM) is a Tl^+^-sensitive fluorescent indicator that is known as a ratiometric Ca2^+^ sensor but is also sensitive to Tl^+^ with different spectral characteristics, preventing signal overlap between these sensitivities. To measure the Tl^+^-BTC complex, its fluorescence is excited at λ_ex_ = 488 nm, and emission is monitored at λ_em_ = 525 nm [65]. This assay was used to study the properties of mitoK_ATP_ [66] and mitoBKCa channels [67] in isolated mitochondria. BTC-AM and another dye with similar properties, Fluozin-2-AM [60], were used by Foster et al. [68] to measure mitochondrial Tl^+^ transport via the ROMK2 channel in permeabilized cells. BTC and Fluozin-2 did not accumulate specifically in mitochondria, and before Foster’s study [68] the latter was used only in high-throughput screening for small molecule inhibitors of plasma membrane-localized ROMK channels [69]. To measure mitochondrial Tl^+^ transport, cells were first loaded with BTC-AM or Fluozin-2-AM. Next, before the Tl^+^ assay, cells were treated with digitonin to permeabilize the plasma membrane, resulting in the loss of the cytosolic component of dye and leaving only the mitochondrial compartment loaded with the indicator. This protocol has previously been used to preferentially localize the above-mentioned PBFI indicator to the mitochondria [64].

One serious disadvantage of using Tl^+^ is the low solubility of thallium chloride (approximately 4 mM), which requires the use of chloride-free solutions. However, a commercially available fluxOR assay based on the same principle but with the fluxOR dye, which is approximately 10-fold more sensitive to thallium than BTC, requires much lower thallium concentrations [70]. This also means that the assay can be carried out in physiological, normal-chloride saline. Unfortunately, the fluxOR assay has never been used in mitochondrial studies.

Several genetically encoded Förster resonance energy transfer (FRET)-based K^+^ indicators have recently been developed [71,126]. It was shown that these indicators could be successfully used for K^+^ fluorescence imaging, which may improve the understanding of intracellular K^+^ signaling pathways. Notably, Bischof et al. [71] designed a genetically encoded potassium ion indicator (GEPII) that was targeted to various intracellular compartments, including mitochondria, and revealed lower K^+^ concentrations ([K^+^]) within mitochondria compared to [K^+^] in the cytoplasm of living cells. Finally, GEPII was used to monitor subcellular K^+^ dynamics during cell depolarization by low extracellular [K^+^]. During such treatment, [K^+^] in cytoplasm decreased while in mitochondria the low [K^+^] gradually increased. Despite these interesting data, the application of these probes to study the function of mitochondrial potassium channels has not yet been demonstrated.

## 5. Biochemical Methods for Studying Mitochondrial K^+^ Channels

Due to the unique properties of mitochondria, it is possible to study K^+^ influx via mitochondrial channels by measuring mitochondrial parameters such as swelling, mitochondrial potential, respiration rate, and mitochondrial ROS synthesis.

Under physiological conditions, mitochondrial volume is regulated by the potassium cycle, i.e., by K^+^ influx and K^+^ efflux via the K^+^/H^+^ antiporter [127]. A slight increase in matrix volume stimulates mitochondrial metabolism through a variety of complex mechanisms [128,129]. Potassium is the most abundant monovalent cation in the cytoplasm, and it mediates changes in volume when the K^+^ conductance of the inner membrane is increased. It has been known for some time that mitochondria may swell in response to valinomycin. This phenomenon is used to measure the activity of mitochondrial potassium channels in isolated mitochondria. The main method for estimating mitochondrial volume is to measure the absorbance of isolated mitochondrial suspensions at 520 nm. Light absorbance decreases with mitochondrial swelling. This kind of simple assay has previously been used to identify the basic properties of mitoK_ATP_ [72] and mitoBK_Ca_ channels [63].

The mitoK_ATP_ channel in brain mitochondria was identified by using the swelling technique [72]. To clarify the role of mitoK_ATP_ in cardioprotective changes in mitochondrial matrix volume, the relationships among mitoK_ATP_, protein kinase, ROS, and mitochondrial permeability transition were measured [73]. The volatile anesthetic sevoflurane increased the cardiac mitochondrial matrix volume by opening the mitoK_ATP_ channel [130]. K^+^ fluxes through cardiac mitoBK_Ca_ channels in the presence of the potassium channel opener NS11021 were studied with light scattering [63].

The mitochondrial swelling method is a simple method, but the final results depend on the complex phenomena of inner mitochondrial membrane permeability, which could be the result of many ion transport pathways being activated. This may cause issues with data misinterpretation by involving specific mitochondrial potassium channels. Moreover, excessive matrix swelling upon calcium ion accumulation occurs because of mitochondrial permeability transition pore (“megachannel”) activation. This process does not involve mitochondrial potassium channels. Hence, we do not recommend this technique for measuring the activity of mitochondrial potassium channels. Complex analysis of the mitochondrial swelling phenomenon has been reviewed recently [131].

K^+^ influx into the mitochondrial matrix is driven by the membrane potential and negatively charged mitochondrial matrix. Hence, mitochondrial potential measurements could be used to track mitochondrial K^+^ channel activity. The influx of K^+^ leads to mitochondrial depolarization, i.e., a decrease in membrane potential. Fluorescent probes such as tetramethylrhodamine methyl ester (TMRM) or ethyl ester (TMRE) are used to measure mitochondrial potential. Depolarization induced by the potassium channel opener NS11021 activating mitoBK_Ca_ in cardiac mitochondria was observed with a TMRM probe [63]. A TMRE probe was used to observe changes in mitochondrial membrane potential due to activation mitoSK_Ca_ channels [77]. The use of fluorescent probes is relatively simple, easy, and useful if the changes in membrane potential are relatively large, which is not obvious given the low density of potassium channels in the inner mitochondrial membrane. TPP^+^ ion-selective electrode is another way to measure changes in membrane potential when used with the isolated mitochondria. This kind of ion-selective electrode was applied to show amoeba [75] and fibroblast [54] mitochondria depolarization due to mitoK_ATP_ channel activation. Using the same method, depolarization because of mitoBK_Ca_ channel activation was observed in potato [22], endothelial [23], *Dictyostelium discoideum* [76], and dermal fibroblast [74] mitochondria. It is important to remember that these kinds of measurements can only be performed on isolated mitochondria.

Mild potassium uncoupling due to K^+^ influx may increase mitochondrial respiration, as observed with the use of classical protonophoric uncouplers. This kind of observation has been reported for a variety of mitochondrial K^+^ channels [54,74]. When mitochondrial channel activation is induced by potassium channel openers, it is critical to show that the increase in mitochondrial respiration has ion selectivity or is inhibited by selective channel blockers. Possible protonophoric uncoupling properties can be ruled out by single patch-clamp recordings.

Due to mild uncoupling caused by K^+^ influx, a decrease in mitochondrial ROS synthesis upon channel activation may also be observed [132]. Interestingly, depolarization of mitochondrial potential due to channel activity may cause an increase in delta pH on the inner mitochondrial membrane [133].

Biochemical studies are useful to characterize mitochondrial potassium channel functions. They help for example to correlate single-channel properties with observations on channel activity related only to mitochondria function such as respiration or high membrane potential. However, there are many disadvantages. For example, mitochondrial depolarization caused by certain drugs could be due to not only the increased influx of K^+^, but also inhibition of the mitochondrial respiratory chain or uncoupling properties of the applied drug. An increase in mitochondrial respiration could be due to K^+^ influx, but it could also be due to the protonophoric properties of the applied drug. In conclusion, biochemical assays for studying mitochondrial K^+^ channel activity are only useful when used in a comprehensive set of experiments.

## 6. Future Membrane Models: Cubic Phases and Solid Supported Membranes

Planar lipid bilayers and patch-clamp techniques described above provide valuable information on the activity of mitochondrial K^+^ channels. The possibility to follow and control single-channel properties is unparalleled. However, there are many disadvantages, such as the randomized orientation of reconstituted channels (PLB), or difficulties in approaching the specific channel, and gigaohm seal formation in the patch-clamp technique. The most pronounced problem is the inherent fragility of the applied membranes, precluding long-term experiments. Additionally, since these techniques rely on a single-molecule approach, individual experiments cannot be converted into the membrane sensing platforms for larger-scale functional studies of drug interactions with mitochondrial K^+^ channels or application in robust analytical devices. To overcome these difficulties, lipid matrices of various topology and geometries can be deposited directly on conducting substrates (electrodes) [134,135]. In such a way, a more stable experimental system can be prepared for the reconstitution of ion channels. Such biomimetic membranes can be investigated using a variety of techniques to describe a structure–function relationship of the embedded membrane proteins: spectroscopic and microscopic techniques including atomic force microscopy or quartz crystal microbalance. Additionally, electrochemistry approaches such as impedance spectroscopy for the evaluation of membrane defectiveness and characterization of membrane capacitive and resistive properties can be applied. This would allow also determining the amount of channel protein reconstituted into the membrane. Here, we will describe the lipidic cubic phase and lipid bilayer solid supported membranes as a potential host for mitochondrial potassium channels in the future.

### 6.1. Lipid Cubic Mesophases as Membranes for Embedding Potassium Channels

**Figure 4:**
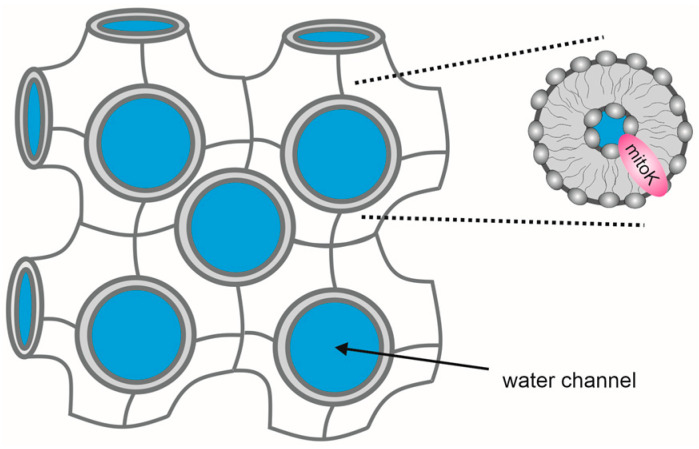
The lipidic liquid crystalline cubic phase (LLC) includes a curved bicontinuous lipid bilayer extending in three dimensions and surrounds two interpenetrating but noncontacting aqueous nanochannels. This double-diamond cubic phase with Pn3m space group shown above is promising for stabilizing membrane proteins and studying their function. Mitochondrial potassium channels could be embedded in the LLC, with their hydrophobic domains incorporated in the lipid bilayer and extramembranous domains exposed to aqueous channels.

Lipid cubic phases (LCPs) consist of a single, continuous lipidic bilayer that forms two interpenetrating but unconnected water networks (Figure 4); they are a promising environment for the reconstitution of membrane proteins that are generally not stable outside of cell membranes but can be stabilized by entrapment in a matrix that resembles a natural environment. Their unique amphiphilic nature allows for the accommodation of hydrophilic substances within aqueous channels, and hydrophobic membrane proteins within the lipid bilayer.

Lipid cubic mesophases coexist with excess water over a wide temperature range and have a flexible structure that can adapt to accommodate the protein. Appropriate amphiphilic compounds with large hydrophilic headgroups such as *n*-octyl-β-d-glucopyranoside are added to increase the aqueous channel sizes for the incorporation of integral membrane proteins with larger extracellular domains. Reversed cubic phases and nanostructured biomimetic materials have been successfully used for the crystallization of several membrane proteins [136,137,138,139,140,141]. LCP technologies for structural studies of membrane proteins such as bacteriorhodopsin or gramicidin were described by Cherezov et al. [142]. The so-called cubicon method for concentrating membrane transporter proteins was described by Ma et al. [143]. The organization of proton pumps and lipids in cubic mesophase crystals was described by Belrhali et al. [144].

LCPs are useful not only for crystallizing membrane proteins and evaluating their structures but also for investigating their properties and functions. For instance, reconstitution and demonstration of enzymatic activity of fructose dehydrogenase (FDH), a membrane-bound redox enzyme, in monoolein LCP was reported [145]. In a spectrophotometric study, it was confirmed that, compared to its behavior in monolinolein-based LCP solutions, in meso immobilized FDH has improved stability [146]. In a recent study, it was shown for the first time that the Na^+^/K^+^-ATPase retained activity when reconstituted in the GMO-based LCP [147]. The reconstitution of bacterial ClC exchanger from *Escherichia coli* in an LCP was described [148], and the efficiency of chloride transport was studied by placing the cubic phase in a lipid membrane device. Active gating in an LCP was described for the glucose transporter protein *Staphylococcus epidermidis* (GlcPSe). Glucose diffusion was tuned by an independent physiological stimulus: the pH gradient [149]. The function of membrane proteins, such as the light-driven bacteriorhodopsin, accommodated in LCPs was also measured [150,151]. Gramicidin dimer crystallization in the cubic mesophase was described [134], and the influence of the lipid used for LCP was investigated [152]. Research into X-ray scattering and circular dichroism revealed that the parameters of the lipid mesophase can change upon peptide addition and that the peptide conformation is dependent on LCP characteristics such as hydrophobic mismatch, the putative lateral pressure profile, and the intrinsic surface curvature of each lipid system. Properties of electrode-supported lipid cubic mesophase films with embedded gramicidin were recently studied [153]. LCPs with embedded proteins can reveal fundamental biological processes and protein–lipid interactions [135,150,154]. They can also be used as a stable nanochannel network for protein chip development [155] or dispersed into nanoparticles such as lyotropic liquid crystalline drug/protein nanocarriers (cubosomes), as discussed in recent reviews [156,157]. LCP presents a new opportunity to study mitochondrial potassium channels; however, its future application requires overcoming several obstacles, including efficient synthesis and functional reconstitution of complex channel proteins.

### 6.2. Solid-Supported and Tethered-Lipid Membranes

**Figure 5:**
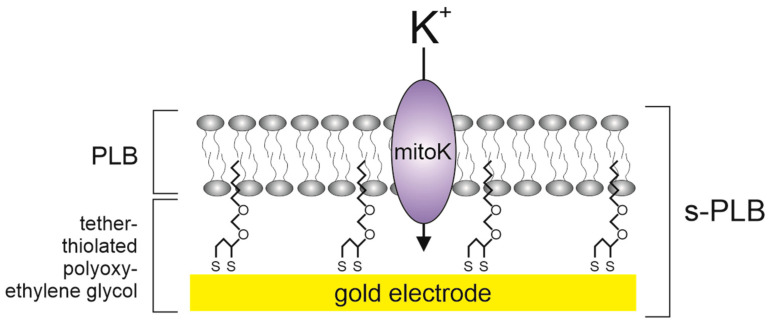
Solid supported membranes. Schematic drawing of the solid planar lipid bilayer s-PLB, showing the following parts: hydrophilic tether (e.g., thiol-derivatized polyoxyethylene glycol), providing covalent binding to the surface of gold, as well as an aqueous layer—a cushion—between the PLB and electrode; mitoK—potassium channel from the inner mitochondrial membrane; PLB—planar lipid bilayer. Please note that this scheme shows only one of the numerous possibilities of s-PLB deposition onto gold electrodes.

Planar solid-supported lipid bilayers (s-PLBs) are model membranes formed on solid surfaces (Figure 5). Similar to PLBs, these structures have been developed as simplified lipid matrices for studies of membrane proteins to model their behavior in a reduced architecture. They provide a controlled environment for functional reconstitution and research on mitochondrial potassium channels. The choice of an electrode in the s-PLB technique usually depends on the signal being measured and the information to be obtained. As such, s-PLBs offer unique opportunities to use a variety of surface-sensitive techniques, particularly dynamic electrochemical techniques, to investigate the charge transfer and separation processes controlled by membrane proteins. In these techniques, the function of ion channels is synonymous with the detection of ion currents.

As described above, free-standing PLBs with large aqueous reservoirs on both sides allow for functional and biophysical studies of ion channels; however, they have the major disadvantage of being extremely fragile and susceptible to mechanical vibrations. Moreover, they have a relatively narrow potential window within which they remain relatively stable, typically up to +/− 100 mV. Depending on the lipid composition and PLB area, the so-called breakdown voltage is close to the limits of the accessible potential range. Therefore, studies on PLBs are limited to this narrow range. In contrast, the s-PLBs formed on metal electrodes allow for the use of a wide range of dynamic electrochemical techniques, including cyclic voltammetry, pulse/differential pulse voltammetry, differential capacitance, chronocoulometry, chronoamperometry, and electrochemical impedance spectroscopy (EIS). However, the major disadvantage of s-PLBs is that by depositing the lipid bilayer on the solid surface, the space available for ion flux between the electrode and the bilayer is reduced. Moreover, the membrane protein structure, particularly the hydrophilic part protruding from the bilayer, can be altered by contact with the metal surface. Therefore, the formation of s-PLBs suitable for membrane protein reconstitution, including mitochondrial potassium channels in a functionally active state, requires that these systems meet a number of criteria:

(a) They should be easily and reproducibly prepared and stable over time;
(b) They should have a lipid bilayer in the liquid crystalline state to allow lateral mobility;
(c) They should have a water reservoir (or, at the very least, a highly hydrated hydrophilic spacer region) between the electrode and the lipid bilayer.

Requirements (b) and (c) are necessary for the incorporation of membrane integral proteins into the lipid bilayer in a functionally active state.

Solid-supported PLBs can be prepared in a variety of ways, generally divided into two groups: bilayers that are physically (electrostatically) adsorbed on a functionalized electrode or chemically (covalently) bound to the surface via a tether chain that connects the lipids to the electrode. The latter case is depicted in Figure 5, with the tether being a polyoxyethylene hydrophilic chain modified on one end with thiol functionality for covalent attachment to metal (gold). The various modes of s-PLB formation are described in more detail in several publications [134,135,152,154] and are brilliantly summarized in the review work [158]. Apart from their stability, the s-PLBs also present another important advantage, which cannot be obtained with the free-standing PLB or patch-clamp technique. They offer the possibility of controlling the electric potential profile across the whole s-PLB. Therefore, it is possible to relate this profile to the protein ion-conducting behavior, including also quantitative identification of ion transfer steps along the channel [159,160,161,162,163,164]. As was already mentioned above, the use of thallium cations as K^+^ surrogates appears to be especially useful in functional studies of potassium channels in whole mitochondria. Along this line, Tl^+^ cations were used in the case of electrode-supported membrane systems to provide a quantitative model of the kinetics, dehydration–hydration, translocation, and charge transfer of this “surrogate” through gramicidin channels through the lipid monolayer deposited on the Hg electrode. The system stability enabled polarization of the monolayer even down to −700 mV. This feature is necessary to utilize the redox behavior of the Tl^+^/Tl^0^ couple in chronoamperometric experiments, expanding the information gained from ion-selective or indirect fluorescent probes. A polarization window larger than typically used in the patch-clamp or free-standing PLB system, and easily achievable in the case of s-PLB, also allows for the evaluation of the channel’s conductivity profile [159,160,165]. Subsequent protein reconstitution into the s-PLBs can be achieved by using the methods typically used for free-standing PLBs, as described above. It is worth noting, however, that the possibility of controlling the polarization of the s-PLB system allows for increasing the effectiveness of channel reconstitution, as was shown in the case of gramicidin A. This feature could be explored in the reconstitution of mitochondrial channels.

This field of research is large and still growing, with studies of channel or pore-forming peptides and proteins in which ion transfer is governed by a passive flux, as well as functional studies on membrane pumps, such as Na^+^/K^+^-ATPases, and proton-pumping ATPases [166,167]. It is worth noting that the use of s-PLBs enables the use of additional non-electrochemical techniques, typically spectroscopic, to strengthen the conclusions drawn from electrochemical experiments. Surprisingly enough, despite many reports on the use of s-PLB in the electrophysiological studies of membrane channels and pumps, to the best of our knowledge, there have been no studies on mitochondrial potassium channels. We think that the use of such model membranes deposited on electrodes can greatly enlarge the information gathered with free-standing planar bilayer membranes. Due to their stability, s-PLBs allow for screening of a large library of ionic species, inhibitors/activators, and other molecules affecting the channel activity in a single set of experiments, using the same s-PLB [158,168]. Successful application of s-PLBs may facilitate studies of mitochondrial potassium channels.

## *7.* Final Remarks

When compared to studies of plasma membrane channels, functional studies of mitochondrial K^+^ channels present new experimental challenges and difficulties. In this review, we focused on experimental troubles and complications with the application of various techniques. We believe that this work will assist researchers in making informed decisions regarding which techniques should be used for optimal experiments, i.e., experiments measuring mitochondrial K^+^ channel activity but not the traffic of other cations. Most likely, the key element of a successful experiment on a given mitochondrial K^+^ channel is an approach that includes multiple techniques. This entails the parallel use of single-channel recordings by electrophysiological techniques and organellar/cellular flux techniques. The latter approach at the cellular level together with an array of biochemical techniques will also help to understand the physiological role of the interaction of mitochondrial K^+^ channels with other proteins.

What techniques do we lack in our methodological portfolio? What technique or techniques will be the most effective in the future? Single K^+^ channel recordings that use patch-clamp or PLB techniques are likely to remain the gold standard in the future. This approach may be supported by new techniques based on solid-supported membranes and lipid cubic phases.

In particular, in our opinion, PLB techniques will see a resurgence in the near future. This is mainly because of the highest (compared to other methods) control of the K^+^ channel reconstitution process in terms of protein channel subunits and the lipid environment. This kind of “experimental control” could be obtained by the application of in vitro translation and nanodisc technology. Additionally, parallel electrophysiological measurements in PLBs combined with single-molecule fluorescence microscopy and high-resolution fluorescence microscopy will add a new dimension to biophysical studies of mitochondrial K^+^ channel function [169,170].

However, credible techniques for measuring mitochondrial K^+^ fluxes in situ, i.e., at the cellular, organ, or animal level, are still lacking. Here, new fluorescent K^+^ sensing probes (small molecules or proteins) are likely to be used for these measurements. Because there are multiple K^+^ transport pathways within mitochondria and cells, the parallel use of specific pharmacological drugs (potassium channel activators or inhibitors) will be required. Powerful chemical synthetic and genetic approaches will aid in solving these issues in the future.

## Figures and Tables

**Table 1:** Overview of methods applied to study mitochondrial potassium channels.

Technique	Parameters Measured	Advantages	Limitations	References
**Electrophysiological Techniques**				
Planar lipid bilayer	Current at a controlled voltage (single-channel conductance, open probability, channel activation/inhibition)	Single-channel measurements—high sensitivity and selectivity, reconstitution into a defined lipid bilayer, easy access to both bilayer sides	Purity of channel protein preparation	[44,45,46,47,48,49,50]
Patch-clamp of the inner mitochondrial membrane	Current at a controlled voltage (single-channel conductance, open probability, channel activation/inhibition)	Single-channel measurements—high sensitivity and selectivity, application to native membranes	Purity of the mitochondria, nonphysiological extramembrane environment	[29,51,52,53,54,55,56,57]
**K^+^ Flux Measurements by Fluorescent Probes**				
Small molecule probes	K^+^ concentration changes	Simple application to cell, isolated mitochondria, or liposomes	Limited localization in mitochondria, poor selectivity for K^+^ over Na^+^, difficult quantification of absolute [K^+^], excitation with phototoxic UV-light	[58,59,60,61,62,63,64]
Tl^+^-sensitive indicators	Tl^+^ concentration changes	Nonfluorescent in the absence of Tl^+^ ions	Low solubility of thallium chloride, nonspecific subcellular localization	[65,66,67,68,69,70]
Genetically encoded probes	K^+^ concentration changes	Specific mitochondrial localization, FRET ratiometric measurements, absolute [K+] determination	Efficient transfection required, sophisticated microscope setup	[71]
**Biochemical Techniques**				
Mitochondrial swelling	Mitochondrial volume	Simple way of macroscopic ion flux measurements, can be measured in fixed cells using electron microscopy	Only isolated mitochondria, low selectivity	[62,63,72,73]
Respiration	Oxygen consumption	Tissue, cells, and isolated mitochondria measurements	Sensitive to nonspecific drug action on mitochondria or drug uncoupling properties	[22,23,53,72,74,75,76]
Mitochondrial potential	Potential changes	Cells and isolated mitochondria	Sensitive to nonspecific action on mitochondria	[22,23,53,74,75,76,77]
